# Population-based cancer incidence in Sikkim, India: report on ethnic variation

**DOI:** 10.1038/bjc.2011.598

**Published:** 2012-01-12

**Authors:** Y Verma, P K Pradhan, N Gurung, S D Sapkota, P Giri, P Sundas, B N Bhattarai, D Nadayil, T Ramnath, A Nandakumar

**Affiliations:** 1 Population Based Cancer Registry, Sir Thutob Namgyal Memorial Hospital, Gangtok, Sikkim, India; 2 National Cancer Registry Programme, Indian Council of Medical Research, Bangalore, India

**Keywords:** incidence, Sikkim, India

## Abstract

**Background:**

A Population-Based Cancer Registry (PBCR) was set up in Sikkim (a state in the North Eastern India) in 2003. We examined incidence rates by ethnic groups from 2003–2008.

**Methods:**

Age-adjusted incidence rates (AARs) per 100 000 person-years were calculated by direct method using the world standard population, and analysed by ethnic group (Bhutia, Rai and other).

**Result:**

There were a total of 1148 male and 1063 female cases of cancer between 2003 and 2008 on the Sikkim PBCR. The overall AARs were 89.4 and 99.4 per 100 000 person-years in males and females, respectively. Incidence rates were highest amongst the Bhutia group (AAR=172.4 and 147.4 per 100 000 person-years in males and females, respectively), and the largest difference in rates were observed for stomach cancers with AARs being 12.6 and 4.7 times higher in the Bhutia group compared with other ethnic groups in males and females, respectively.

**Conclusion:**

These observations call for further epidemiological investigations and the introduction of screening programmes.

The Population-Based Cancer Registry (PBCR) in Sikkim was started in 2003 under the National Cancer Registry Programme (NCRP) of the Indian Council of Medical Research (ICMR). The initiative by the ICMR to commence six PBCRs in the North Eastern states of India was due to the possible higher incidence of cancer reported under the project on development of an atlas of cancer in India (Nandakumar *et al*, 2005). Cancer registration as in the other PBCRs in India is active.

Cancer of the stomach among migrant ethnic groups has been studied in Illinois, USA (Cho *et al*, 1996). Similarly, American ethnic groups like the American Indians and Alaska Natives have shown greater risk for stomach cancers than the non-Hispanic Whites (Wiggins *et al*, 2008).

There is paucity of data on the prevalence of oesophageal cancer risk factors by race, ethnicity and gender. However, a study showed that age-adjusted oesophageal cancer incidence rates in blacks was more than twice the rate in whites (8.63 *vs* 4.39, *P*<0.05) (Baquet *et al*, 2005).

In a study conducted among specific American Pacific Islander population in the US, it was found that incidence of liver cancer among Samoan men was the highest (Miller *et al*, 2008).

The incidence rates of nasopharyngeal cancer are as high as 300–800 cases per 1 million in some Cantonese regions of Southern China (Muir *et al*, 1987).

In India, however, no study has been done on ethnic populations with reference to incidence of stomach, oesophageal, nasopharyngeal, and liver cancer.

In Sikkim, there are 13 ethnic groups having diverse lifestyle and dietary habits. The Bhutias were early immigrants from the Tibetan province of Khams (Risley, 1928). The dominant heterogeneous Nepalese population of various ethnicities, including the Rais, are later immigrants from Nepal (Lama, 1994; Menon and Banerjea, 2005). We report the incidence and patterns of cancer including the ethnic variation observed during the first 6 years (2003–2008) of registry operation.

## Materials and methods

The PBCR covers the entire state of Sikkim, with a population of 540 851 (2001 census of India). A standard incidence and mortality form by the NCRP is used to collect the respective information. This form has set guidelines and is followed by all 24 PBCRs within the NCRP network. The third edition of the International Classification of Diseases for Oncology (ICD-O-3) was used to classify tumours, and only malignant neoplasms (those with behaviour code 3) are included in the registry (April *et al*, 2000).

The registry population area at risk was estimated using the 1991 and 2001 census population (Census of India 1991, 2001) by sex, as well as the growth rate during that interval using the difference distribution method (Takiar and Shobana, 2009). The population estimation according to ethnic group was estimated using the 2001 census population and ethnic breakup of the population provided by Directorate of Economics, Statistics, Monitoring and Evaluation (DESME) (State Socioeconomic census, 2006). The 13 ethnic groups as defined by DESME were aggregated into the following 3 main groups due to small numbers of cases: Bhutia, Rai and others (including all 11 other ethnic groups). The age-adjusted incidence rates (AAR) per 100 000 person-years, were calculated by direct method using the world standard population (Jensen *et al*, 1991).

## Results

There were 2211 registered cancer cases during the 6-year period, 2003–2008. The average annual AAR per 100 000 population for all sites was 89.4 in males and 99.4 in females. The most common site of cancer in males was stomach cancer that comprised 15% of all sites of cancer, followed by cancer of the oesophagus (10%). In females, the most common site of cancer was cancer of the cervix uteri accounting for 11.7% of all cancers in females, followed by cancer of the breast (9.1%). Among females, cancers of the stomach and oesophagus were also among the leading sites of cancer 8.1% (AAR-8.75) and 7.6% (AAR-7.5), respectively.

The average AAR of all sites of cancer combined in both males and females are slightly lower than the urban registries and higher than that of the only rural registry data of Barshi. However, when the AARs of specific sites of cancer are examined, higher incidence rates are seen in cancers of the stomach, oesophagus, and nasopharyngeal cancer in both sexes (National Cancer Registry Programme. Indian Council of Medical Research, 2010).

Table 1 gives the distribution of the number of cases by sex and ethnic group, alongside the population at risk in each category. The Bhutia group comprised approximately 13% of the population but accounted for almost double the proportion of cancers.

Table 2 gives the AAR for all sites and leading sites of cancer in both males and females by ethnic groups. The AAR in Bhutia males was 172.4 per 100 000 compared with 72.9 in the Rai group and 76.8 in the ‘others’ category. Likewise, among females the AAR for all sites of cancer was 147.4 compared with 111.9 and 87.8 for Rai and the ‘others’ group, respectively. Among the leading sites of cancer, cancer of the stomach showed the most significant difference between the Bhutia and the other two groups in both males and females. The AARs among the Bhutias was 12.6 and 4.7 times higher than the Rai group in males and females, respectively.

Supplementary Table III gives the number of cancers (all ages), relative frequencies (%), average annual crude, age-adjusted incidence rates (AAR), and proportion of microscopic verification (%) by site (ICD-10) and sex (2003–2008) of the PBCR, Sikkim. Supplementary Table IV gives the comparison of age-adjusted incidence rates (AAR) of common anatomical sites of cancer of PBCR, Sikkim with older PBCRs in India. Supplementary Figure 1 shows the comparison of AAR of stomach cancer of Sikkimese Bhutia population (2003–2008) with that of other countries (2008). Supplementary Figure 2 shows the comparison of AAR of oesophageal cancer of Sikkimese Bhutia population (2003–2008) with that of other countries (2008).

## Discussion

Data on cancer patterns helps in determining the strategies for cancer control and prevention in different countries. It provides important information on the risk of different cancers especially in vulnerable ethnic groups in the population and is important in planning and evaluation of cancer prevention and early detection.

The average annual AARs of cancers of all anatomical sites in both males and females are lower than the older urban PBCRs of India but higher than the only rural registry of Barshi. The state of Sikkim covers both urban and rural areas. However, when site-specific comparison of AARs is done the AARs of both stomach and oesophageal cancers are higher in both sexes across all the other six PBCRs. Further breakdown of the AARs by ethnic group reveals that these rates for both stomach and oesophageal cancers are several fold higher than not only the other PBCRs but also that of the rest of the population of Sikkim. Stomach cancer AARs of 60.4 and 29.4 per 100 000 in males and females, respectively, of the Bhutia population is also higher than that of the highest AAR (55.4 in males and 24.4 in females) in Aizawl district reported among the PBCRs in India during this time period (National Cancer Registry Programme. Indian Council of Medical Research, 2010). International comparisons also show that the AARs of stomach cancer are higher than that of the rates in Japanese populations that have since many years reported the highest AARs in the world (Ferlay *et al*, 2010).

Such high rates of stomach cancer could be due to the fact that majority of the population of Sikkim consumes fermented foods like *gundruk* (dried and decayed leaves of mustard oil plant and spinach)*, sinki* (radish that is decayed and dried) and *kinema* (fermented soya beans). The population, especially the Bhutias residing in rural areas, also consume salted and smoked fried meat. Alcohol drinking and tobacco smoking is also very prevalent among them. Consumption of country-made wine, also known as *chang* (local wine prepared by fermenting millet using yeast), along with dried smoked preserved meat is an age-old tradition especially in the Bhutia tribes. So far no study has investigated on prevalence of *Helicobacter pylori* infestation in Sikkim or the Bhutia population.

Ethnic population studies can give an insight into the type of cancers prevalent in that ethnic group. It is essential that in countries with a high incidence of stomach cancer, primary prevention should be promoted to reduce the disease burden. Testing and treatment of *H*. pylori in high incidence areas should be complemented with dietary modifications (Graham, 2000).

Prognosis for stomach cancer patients identified by screening may be better than for those identified by other means (Hanazaki *et al*, 1997). The advantage of this is that screening programmes and interventions can be planned accordingly in a focused population and bring down the cost drastically. This is especially very relevant in a low resource setting. However, this study also suggests the importance of undertaking case–control studies to look for the causes and vulnerability of the Bhutia ethnic group to cancer of the stomach.

Similar higher AAR is seen among men and women for cancer of oesophagus especially in the Bhutia population (male AAR-22.6 and female AAR-13.1). In India the incidence of oesophageal cancer is moderately high. Oesophageal cancer is the second most common cancer among males and the fourth most common cancer among females according to combined data from cancer registries in India (Gajalakshmi *et al*, 2001). Various foods and food additives have been studied for their association with this disease (Phukan *et al*, 2001).

Cancer of the nasopharynx in Bhutia males shows an AAR of 5.9 as compared with the AAR of 3.3 in males in entire Sikkim. This is higher than the AAR of the other countries and India. Consumption of salted fish, including partial fermentation and nitrosamine formation, has been especially associated with increased risk of nasopharyngeal cancer in South East Asia (IARC Monographs, 1993).

Liver cancer is a major health problem in developing countries. The highest incidences are recorded in China, Japan, and South East Asia. In both high and low incidence areas there is great variability in incidence among ethnic groups (Engstrom *et al*, 1997). In Bhutia males the AAR was 19.6 and in Bhutia females 8.1, which are comparatively higher than the other ethnic groups residing in Sikkim.

This is the first report of this kind after the establishment of PBCR in 2003. The ethnic variations bring to light the incidence and pattern of cancer of the stomach, oesophagus, liver, and nasopharynx among the Bhutia ethnic group of Sikkim. This gives an opportunity to take up case–control studies in the future. Apart from this the report indicates the need to take up screening programmes in the Bhutia population for cancers of the stomach (Miyamoto *et al*, 2007; Yoshihara *et al*, 2007).

## Figures and Tables

**Table 1:** Population at risk and cancer cases based on ethnic groups of Sikkim (2003–2008)

**Sikkim**	**Population at risk**				**Cases**			
**ethnicity**	**Male**	**%**	**Female**	**%**	**Male**	**%**	**Female**	**%**
Bhutia	255 538	12.8	231 495	13.3	305	26.6	238	22.4
Rai	264 469	13.3	240 811	13.8	139	12.1	162	15.2
Others	147 239 4	73.9	126 716 9	72.8	704	61.3	663	62.4
All Sikkim	199 240 1	100	173 947 5	100	1148	100	1063	100

**Table 2:** Leading sites of cancer by ethnic groups of Sikkim giving number (No) of cases and the age-adjusted incidence rates (AAR) per 100 000 population (2003–2008)

**Bhutia**			**Rai**			**Others**		
**Site**	**No**	**AAR**	**Site**	**No**	**AAR**	**Site**	**No**	**AAR**
*Males*								
Stomach	104	60.6	Lung and others	14	8.2	Oesophagus	66	7.6
Oesophagus	38	22.6	Oesophagus	11	6	Stomach	59	6.8
Liver	34	19.6	Nasopharynx	10	5.2	Lung and others	57	6.8
Lung and others	14	8.6	Liver	10	5.1	Liver	50	5.8
Nasopharynx	12	5.9	Stomach	9	4.8	Larynx	47	5.4
Other skin	8	3.9	Brain, nervous system	7	3.2	Mouth	29	3.1
Larynx	7	3.7	Larynx	7	4.8	Nasopharynx	28	2.5
Colon	6	3.3	Hypopharynx	6	3.9	Brain, nervous system	27	2.5
Brain, nervous system	5	1.3	Rectum	5	2.7	Hypopharynx	26	3.2
Tongue	5	3.0	Mouth	4	2.5	Myeloid leukaemia	19	1.5
All sites	305	172.4	All sites	139	72.9	All sites	705	76.8
								
*Females*								
Stomach	43	29.4	Breast	19	12.1	Cervix Uteri	88	10
Cervix uteri	23	13.2	Lung and others	17	14.1	Breast	61	7.8
Oesophagus	22	13.1	Cervix uteri	12	8	Oesophagus	50	6.8
Breast	17	9.5	Oesophagus	9	6.3	Lung and others	45	7.2
Liver	14	8.1	Stomach	8	6.2	Stomach	35	4.8
Gallbladder and others	13	8.7	Liver	8	6.3	Ovary and others	29	3.5
Lung and others	11	6.8	Gallbladder and others	7	5.9	Liver	29	3.9
Ovary and others	10	6.4	Larynx	7	4.7	Gallbladder etc.	27	4
Myeloid leukaemia	8	3.8	Bladder	6	5.1	Larynx	22	3.1
Other skin	8	5.3	Other skin	5	4.2	Brain, Nervous Sys.	21	2.6
All sites	238	147.4	All sites	162	111.9	All Sites	662	87.9
