# Supplementary material for: Population-based cancer incidence in Sikkim, India: report on ethnic variation
Source: Br J Cancer. 2012 Jan 12;106(5):962–5. doi: 10.1038/bjc.2011.598 (PMC3305974; doi:10.1038/bjc.2011.598)
Supplement: Supplementary Information [file bjc2011598x1.doc]

***Supplementary Online Material:***

1. Table III: Number of cancers (No.) (all ages) Relative Frequencies (%), Average Annual Crude (CR), Age Adjusted Incidence Rates Rates (AAR) and proportion of Microscopic Verification (MV %) by Site (ICD-10) and Sex (2003-2008)
2. Table IV: Comparison of Age Adjusted Incidence Rates (AAR) of common anatomical sites of cancer with older PBCRs in India.
3. Figure 1: Comparison of AAR of Stomach Cancer of Sikkimese Bhutia Population (2003-2008) with that of other Countries (2008)
4. Figure 2: Comparison of AAR of Oesophageal Cancer of Sikkimese Bhutia Population (2003-2008) with that of other Countries (2008)

**Supplementary Online material**

Table III: Number of cancers (No.) (all ages) Relative Frequencies (%), Average Annual Crude (CR), Age Adjusted Incidence Rates Rates (AAR) and proportion of Microscopic Verification (MV %) by Site (ICD-10) and Sex (2003-2008)

| **ICD-10** | **SITE** | **MALES** | | | | | **FEMALES** | | | | |
| --- | --- | --- | --- | --- | --- | --- | --- | --- | --- | --- | --- |
|  |  | **No** | **%** | **CR** | **AAR** | **MV %** | **No** | **%** | **CR** | **AAR** | **MV %** |
| **C00-06** | **Oral Cavity** | 67 | 5.8 | 3.4 | 5.2 | 85.1 | 29 | 2.7 | 1.7 | 2.9 | 79.3 |
| **C07-08** | **Salivary Gland** | 4 | 0.3 | 0.2 | 0.2 | 50.0 | 9 | 0.8 | 0.5 | 0.6 | 88.9 |
| **C09-10,11-14** | **Pharynx** | 107 | 9.3 | 5.4 | 8.1 | 86.0 | 51 | 4.8 | 2.9 | 4.9 | 88.2 |
| **C15** | **Oesophagus** | 115 | 10.0 | 5.8 | 9.5 | 73.0 | 81 | 7.6 | 4.7 | 7.5 | 65.4 |
| **C16** | **Stomach** | 172 | 15.0 | 8.6 | 14.2 | 68.6 | 86 | 8.1 | 4.9 | 8.8 | 77.9 |
| **C17** | **Small Intestine** | 3 | 0.3 | 0.2 | 0.2 | 33.3 | 0 | 0 | 0 | 0 | - |
| **C18** | **Colon** | 15 | 1.3 | 0.8 | 1.2 | 60.0 | 12 | 1.1 | 0.7 | 1.1 | 83.3 |
| **C19-21** | **Rectum and Anal canal** | 28 | 2.4 | 1.4 | 2.1 | 60.7 | 11 | 1 | 0.6 | 1.1 | 63.6 |
| **C22** | **Liver** | 94 | 8.2 | 4.7 | 7.6 | 59.6 | 51 | 4.8 | 2.9 | 4.9 | 35.3 |
| **C23-24** | **Gallbladder etc.** | 18 | 1.6 | 0.9 | 1.5 | 44.4 | 47 | 4.4 | 2.7 | 4.9 | 48.9 |
| **C25** | **Pancreas** | 14 | 1.2 | 0.7 | 1.1 | 14.3 | 9 | 0.8 | 0.5 | 0.8 | 33.3 |
| **C30-31** | **Nose,Sinuses etc.** | 5 | 0.4 | 0.3 | 0.4 | 80.0 | 8 | 0.8 | 0.5 | 0.9 | 75.0 |
| **C32** | **Larynx** | 61 | 5.3 | 3.1 | 4.9 | 65.6 | 30 | 2.8 | 1.7 | 3 | 66.7 |
| **C33-34** | **Lung etc.** | 85 | 7.4 | 4.3 | 7.4 | 44.7 | 73 | 6.9 | 4.2 | 8 | 41.1 |
| **C37-38** | **Other Thoracic Organs** | 4 | 0.3 | 0.2 | 0.3 | 75.0 | 1 | 0.1 | 0.1 | 0.1 | 100.0 |
| **C40-41** | **Bone** | 19 | 1.7 | 1 | 1.1 | 78.9 | 9 | 0.8 | 0.5 | 0.7 | 33.3 |
| **C43-46** | **Skin etc.** | 34 | 3.0 | 1.7 | 2.9 | 91.2 | 30 | 2.8 | 1.7 | 3.2 | 86.7 |
| **C47:49** | **Conn.&Soft Tissue** | 12 | 1.0 | 0.6 | 0.7 | 75.0 | 9 | 0.8 | 0.5 | 0.7 | 77.8 |
| **C50** | **Breast** | 4 | 0.3 | 0.2 | 0.3 | 100.0 | 97 | 9.1 | 5.6 | 8.5 | 91.8 |
| **C53** | **Cervix** | - | - | - | - | - | 124 | 11.7 | 7.1 | 10.1 | 87.9 |
| **C51-52;54;57-58** | **Other female genitals** | - | - | - | - | - | 25 | 2.4 | 1.4 | 2.3 | 80.0 |
| **C55** | **Uterus unspecified** | - | - | - | - | - | 6 | 0.6 | 0.3 | 0.6 | 50.0 |
| **C56** | **Ovary** | - | - | - | - | - | 44 | 4.1 | 2.5 | 3.7 | 65.9 |
| **C61** | **Prostate** | 13 | 1.1 | 0.7 | 1.2 | 23.1 | - | - | - | - | - |
| **C62;60;63** | **Other male genitals** | 16 | 1.4 | 0.8 | 1 | 93.8 | - | - | - | - | - |
| **C67** | **Bladder** | 22 | 1.9 | 1.1 | 1.8 | 68.2 | 12 | 1.1 | 0.7 | 1.2 | 41.7 |
| **C64-66;68** | **Kidney** | 9 | 0.8 | 0.5 | 0.6 | 55.6 | 5 | 0.5 | 0.3 | 0.5 | 20.0 |
| **C69** | **Eye** | 1 | 0.1 | 0.1 | 0 | - | 2 | 0.2 | 0.1 | 0.2 | 50.0 |
| **C70-72** | **Brain, Nervous System** | 39 | 3.4 | 2 | 2.5 | 5.1 | 30 | 2.8 | 1.7 | 2.7 | 13.3 |
| **C73** | **Thyroid** | 18 | 1.6 | 0.9 | 1.4 | 88.9 | 21 | 2 | 1.2 | 2.1 | 100.0 |
| **C74** | **Adrenal Gland** | 0 | 0.0 | 0 | 0 | - | 3 | 0.3 | 0.2 | 0.2 | 33.3 |
| **C81** | **Hodgkin’s Disease** | 4 | 0.3 | 0.2 | 0.3 | 75.0 | 7 | 0.7 | 0.4 | 0.5 | 100.0 |
| **C82-85,96** | **Non-Hodgkinslymphoma** | 10 | 0.9 | 0.5 | 0.6 | 80.0 | 10 | 0.9 | 0.6 | 0.9 | 100.0 |
| **C88,90** | **Multiple Myeloma** | 6 | 0.5 | 0.3 | 0.5 | 83.3 | 7 | 0.7 | 0.4 | 0.7 | 42.9 |
| **C91** | **Lymphoid leukaemia** | 6 | 0.5 | 0.3 | 0.3 | 100.0 | 6 | 0.6 | 0.3 | 0.4 | 100.0 |
| **C92-93;95** | **Myeloid leukaemia** | 29 | 2.5 | 1.5 | 1.5 | 86.2 | 28 | 2.6 | 1.6 | 2.1 | 82.1 |
| **Others and unspecified** | | 114 | 9.9 | 5.7 | 9.1 | 29.8 | 90 | 8.5 | 5.2 | 8.8 | 37.8 |
| **All Sites but skin** | | 1119 | 97.5 | 56.2 | 87 | 62.8 | 1034 | 97.3 | 59.4 | 96.3 | 67.1 |
| **All Sites (C00 – C96)** | | **1148** | **100.0** | **57.6** | **89.4** | **63.5** | **1063** | **100.0** | **61.1** | **99.4** | **67.6** |

Table IV: Comparison of Age Adjusted Incidence Rates (AAR) of common anatomical
sites of cancer with older PBCRs in India.

| **SITE** | **Sikkim**  **(2003-2008)** | **Bangalore**  **(2006-2007)** | **Barshi**  **(2006-2008)** | **Bhopal**  **(2006-2008)** | **Chennai**  **(2006-2008)** | **Delhi**  **(2006-2007)** | **Mumbai**  **(2006-2008)** |
| --- | --- | --- | --- | --- | --- | --- | --- |
| **Males** |  |  |  |  |  |  |  |
| Stomach | 14.4 | 9.5 | 2.1 | 2.8 | 11.8 | 3.5 | 4.3 |
| Oesophagus | 9.5 | 7.9 | 3.4 | 6.0 | 7.7 | 4.6 | 4.8 |
| Lung | 7.3 | 10.7 | 2.7 | 11.8 | 13.0 | 13.9 | 9.6 |
| Larynx | 5.1 | 3.7 | 2.2 | 5.5 | 4.3 | 8.0 | 5.1 |
| Nasopharynx | 3.3 | 0.3 | 0.1 | 0.2 | 0.7 | 0.5 | 0.4 |
| All Sites | 89.4 | 113.4 | 51.5 | 104.6 | 115.2 | 124.3 | 99.1 |
| **Females** |  |  |  |  |  |  |  |
| Cervix uteri | 10.2 | 21.1 | 18.6 | 18.9 | 18.5 | 17.9 | 14.1 |
| Breast | 8.7 | 36.1 | 8.8 | 25.4 | 31.5 | 32.3 | 32.3 |
| Stomach | 9 | 5.6 | 2.3 | 1.5 | 6.0 | 1.4 | 2.7 |
| Oesophagus | 7.7 | 6.4 | 2.2 | 5.4 | 4.3 | 2.8 | 2.9 |
| Lung | 8.1 | 3.9 | 2.4 | 2.7 | 4.1 | 3.6 | 3.8 |
| All Sites | 99.4 | 139.1 | 55.1 | 105.5 | 121.1 | 121.2 | 110.4 |

Figure 1: Comparison of AAR of Stomach Cancer of Sikkimese Bhutia Population

(2003-2008) with that of other Countries (2008)


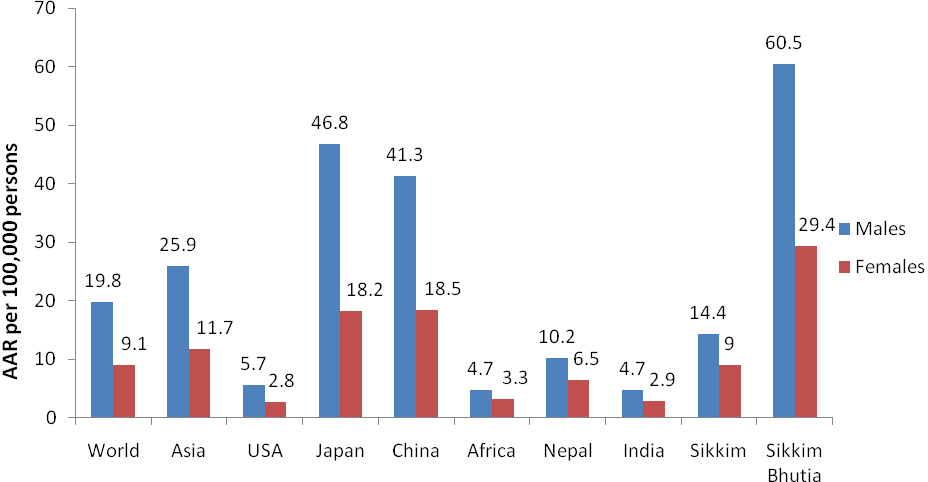


Figure 2: Comparison of AAR of Oesophageal Cancer of Sikkimese Bhutia Population (2003-2008) with that of other Countries (2008)
